# Two-staged hybrid repair of multiple great vessel and thoracic aneurysms with right vocal fold palsy using retrograde in situ branched stent grafting: a case report

**DOI:** 10.1186/s44215-024-00142-w

**Published:** 2024-03-04

**Authors:** Yuki Imamura, Yoshiaki Saito, Kenyu Murata, Rin Itokawa, Ryo Taguchi, Masahito Minakawa

**Affiliations:** Department of Thoracic and Cardiovascular Surgery, Hirosaki University School of Medicine, 5 Zaifu-cho, Hirosaki, Aomori, 036-8562 Japan

**Keywords:** Retrograde in situ branched grafting, Multiple aneurysms, Two-stage repair

## Abstract

**Background:**

Repair of multiple thoracic aneurysms with vocal fold palsy represents a surgical challenge. Here, we present a case of multiple cervical and thoracic aortic aneurysms with right vocal fold palsy successfully treated with two-stage hybrid repair using retrograde in situ branched grafting.

**Case presentation:**

A 52-year-old patient with multiple cervical and thoracic aortic aneurysms and right vocal fold palsy was treated using two-stage hybrid repair. Initially, the patient underwent an open partial arch replacement with reconstruction of the right subclavian, right common carotid, and left common carotid arteries. Three months after the initial procedure, the left subclavian artery was reconstructed during thoracic endovascular aortic repair with retrograde in situ stent grafting using the needle technique. The postoperative course was uneventful with no endoleaks detected using postoperative computed tomography.

**Conclusion:**

This case highlighted the potential of two-stage repair of multiple thoracic aneurysms using retrograde in situ stent grafting. With the expansion of surgical options to include endovascular and hybrid approaches, surgical procedures should be carefully planned for individuals with complex aortic diseases.

**Supplementary Information:**

The online version contains supplementary material available at 10.1186/s44215-024-00142-w.

## Background

Multiple thoracic aneurysm repair has rarely been reported. Total arch replacement is the first-line treatment for aortic arch aneurysms; however, recurrent laryngeal nerve injury is a well reported and serious complication [1]. Although thoracic endovascular aortic repair (TEVAR) for aortic arch aneurysms is less invasive, it is challenging because of the need for cervical vessel reconstruction. Therefore, in this report, we present a case of multiple cervical and thoracic aortic aneurysms with right vocal fold palsy successfully treated with two-stage hybrid repair using retrograde in situ branched grafting (RIBS). The patient provided written informed consent for the report of his case details and imaging studies.

## Case presentation

**Fig. 1:**
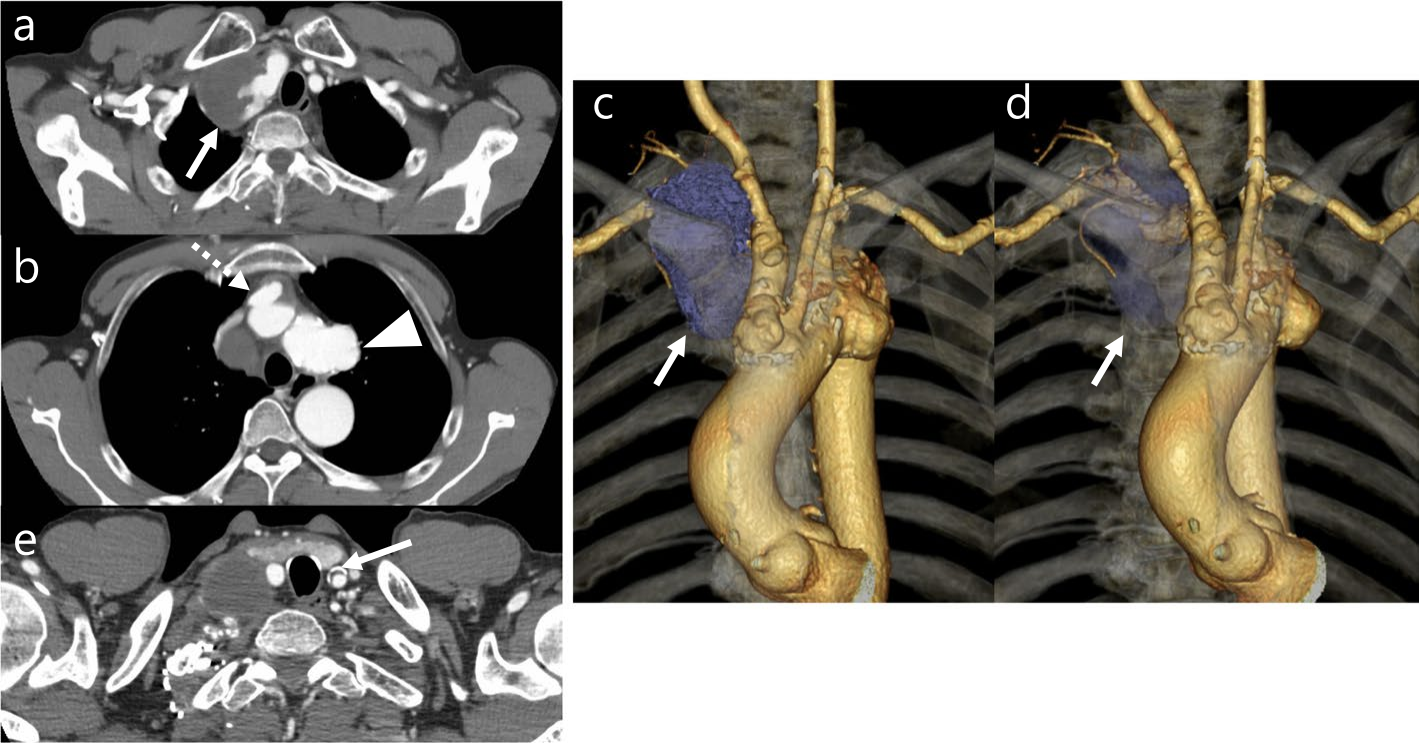
**a, b** Preoperative computed tomography scan revealing aneurysms of right subclavian artery (solid line) and brachiocephalic artery (dotted line) and saccular aortic arch aneurysms (arrow head). **c, d** Preoperative three-dimensional computed tomography scan revealing right subclavian artery aneurysm (blue color lesion, arrow head). **e** Preoperative computed tomography scan revealing moderate plaque in the left common carotid artery (solid line)

**Fig. 2:**
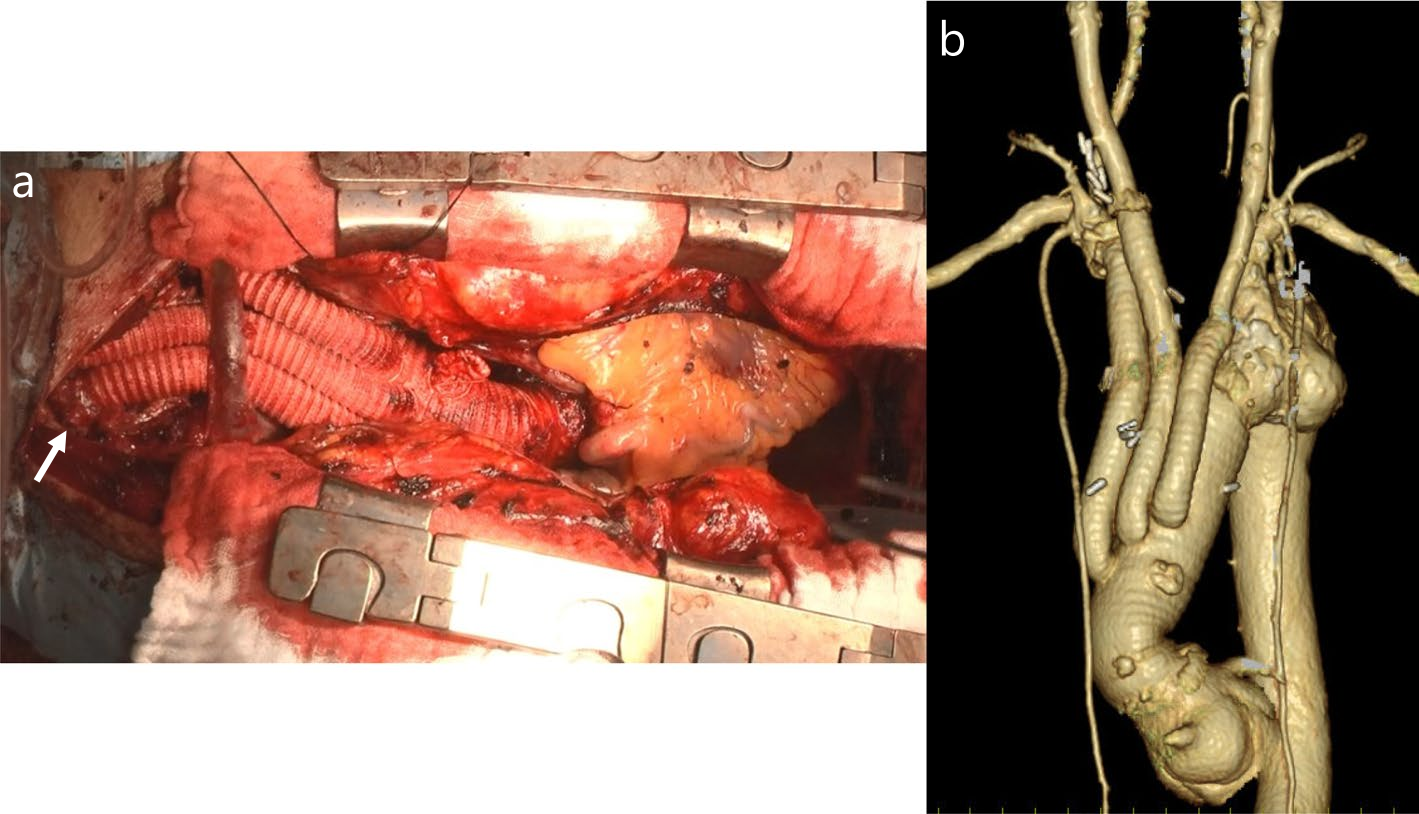
**a** Intraoperative image showing the right subclavian artery anastomosis site (arrow head). **b** Postoperative three-dimensional computed tomography image of the initial open procedure

**Fig. 3:**
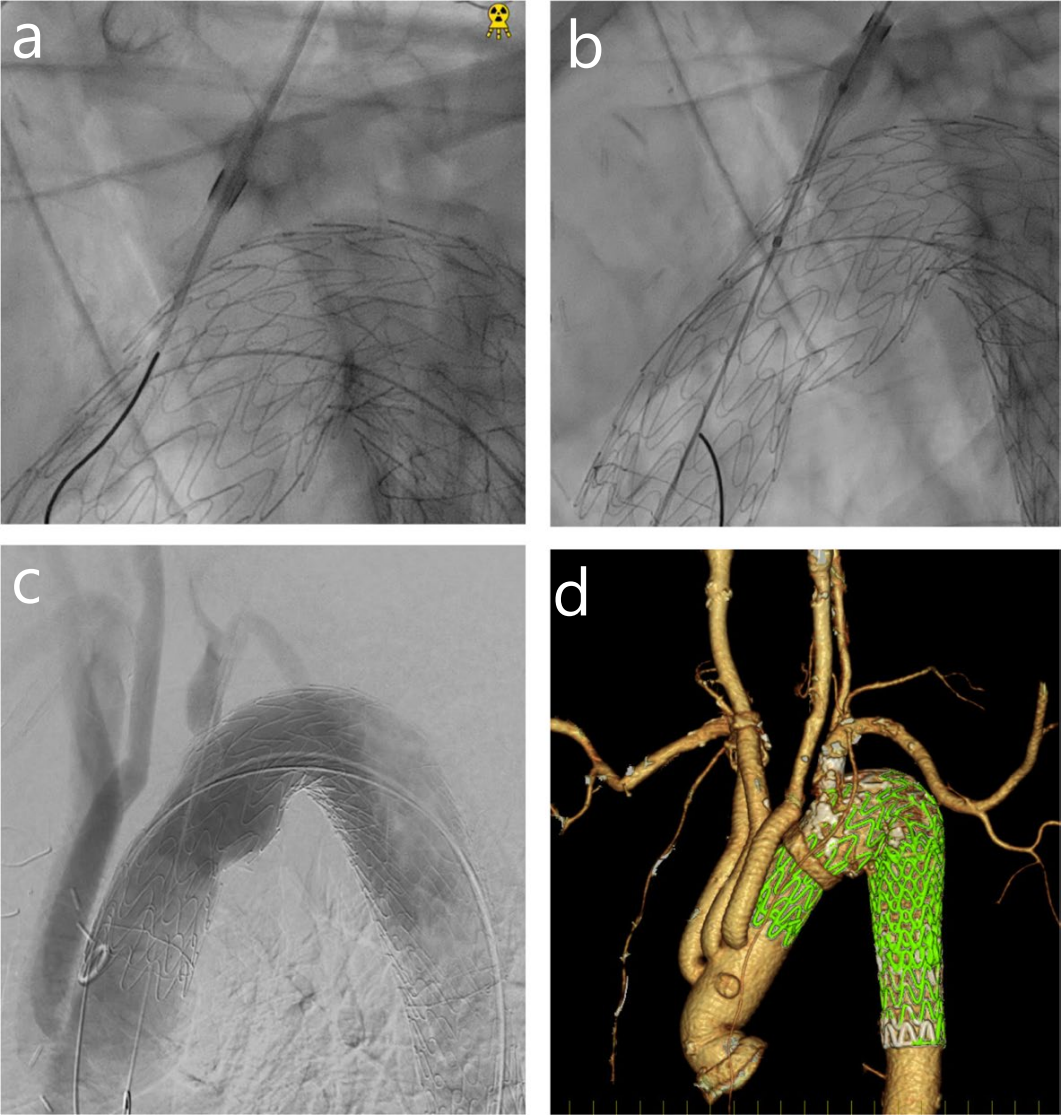
**a** An 18-guage percutaneous transhepatic gallbladder drainage needle advanced into the stent graft. **b** An 11–29-mm Viabahn endoprosthesis advanced into the stent graft, and the puncture hole was deployed. **c** Post-surgery angiography. **d** Postoperative three-dimensional computed tomography image

A 52-year-old man with new-onset hoarseness since 6 months was diagnosed with vocal fold mobility impairment secondary to right recurrent laryngeal nerve palsy. Contrast-enhanced computed tomography (CT) revealed aneurysms in the right subclavian artery (RSCA) and brachiocephalic artery (BCA), as well as saccular aortic aneurysms (Fig. 1a–d), with maximum diameters of 43 mm, 40 mm, and 42 mm, respectively; therefore, surgery was recommended. Moreover, 30 years earlier, he had undergone radiation therapy for a cervical tumor; however, no information was available on the dose and extent of the radiation administered. There were no features suggestive of radiation dermatitis. Thus, the patient would have been at a high risk for bilateral recurrent laryngeal nerve palsy during manipulation of the distal aortic arch and left subclavian artery (LSCA). Moreover, preoperative CT (Fig. 1e) revealed a moderate plaque in the left common carotid artery (LCCA). Cerebral magnetic resonance imaging visualized equal-sized vertebral arteries. The surgical plan included a hybrid repair with an initial open partial arch replacement and reconstruction of the RSCA, right common carotid artery (RCCA), and LCCA, followed by TEVAR using RIBS. Furthermore, median sternotomy was used for partial arch replacement. Adhesion between the brachiocephalic vein and artery was severe during surgery. Additionally, open distal anastomosis was used for partial arch replacement using a 26-mm quadrifurcated graft (J Graft Vascular Prosthesis, Japan Lifeline, Tokyo, Japan) with antegrade cerebral perfusion. Subsequently, 12-F balloon-tipped cannulas (Senko Medical Instrument, Japan) were inserted into the LSCA, LCCA, RCCA, and RSCA. The arch vessels (RSCA, LCCA, and RCCA) were individually reconstructed (Fig. 2a). The initial postoperative course was uneventful, and postoperative CT revealed excellent graft patency (Fig. 2b). Histopathological examination revealed atherosclerosis in the RSCA and BCA aneurysms. Three months after the initial operation, the RIBS technique was used for LSCA reconstruction during TEVAR. During the procedure, the LSCA and right common femoral artery were exposed. Then, a 9-Fr short sheath (Brite tip, Cordis, Miami, FL, USA) was inserted from the LSCA, and a 5-Fr Fogarty catheter (Edwards Lifesciences Co., Irvine, CA, USA) was used for temporary LSCA orifice occlusion to prevent embolic stroke to the vertebral artery. Next, the 31-26-100-mm Gore TAG Conformable Thoracic Stent Graft with Active Control System (C-TAG with ACS; W. L. Gore & Associates, Flagstaff, AZ, USA) was used with its proximal end aligned 2 cm distal to the arch aneurysm. The 34–200-mm conformable TAG was then deployed at the aortic arch protruding at least 4 cm from the aortic arch saccular aneurysm, and the 5-Fr Fogarty catheter was deflated. The steep angle between LSCA and the aortic arch was approximately 45°. Subsequently, a manually bent percutaneous transhepatic gallbladder drainage needle (18 gauge × 200 mm; Hanaco Medical, Saitama, Japan) at approximately 45° was used to puncture the stent graft (Fig. 3a), followed by dilation of this stent graft puncture site using a high-pressure Conquest balloon (6-20 mm) (Bard Peripheral Vascular). Next, the LSCA was reconstructed with an 11–29-mm Viabahn endoprosthesis (W. L. Gore & Associates) (Fig. 3b), and a 10-25-mm balloon-expandable Express LD stent (Boston Scientific Corp, Natick, MA, USA) was deployed at the junction of the main stent and branched stent graft to reinforce the fixation and prevent type III endoleaks. Final angiography confirmed that the arch aneurysm was completely excluded without endoleaks (Fig. 3c). The complete procedures are presented in the Video. The postoperative course was uneventful, and postoperative CT revealed excellent graft patency with no endoleaks (Fig. 3d). The patient had no complications, cerebral ischemia, or endoleaks 15 months postoperatively.

## Discussion and conclusions

This report describes the management of multiple aneurysms with vocal fold mobility impairment secondary to right recurrent laryngeal nerve palsy using two-stage hybrid repair. An alternative surgical plan is total arch replacement with a commercially available frozen elephant trunk (FET), facilitating a one-stage repair. However, it is unsuitable for type III arches [2, 3] due to the possibility of kinking and the spring-back phenomenon unique to FET causing distal stent graft-induced new entry (d-SINE). Moreover, manipulation around the patient’s LSCA and distal aortic arch would be needed because of the high degree of adhesion as a result of post-radiation therapy, resulting in a higher risk of left recurrent laryngeal nerve injury. Therefore, a hybrid two-staged procedure with an initial open partial arch replacement followed by TEVAR was considered. The necessity of preserving the LSCA has increasingly been described [4]. We had initially planned to perform the LCCA to LSCA bypass. However, as a moderate atherosclerotic lesion was detected in this patient at the LCCA anastomosis site during the carotid-subclavian bypass, we selected initial open partial arch replacement followed by the RIBS technique for reconstruction of the LSCA during TEVAR. Our surgical plan facilitated manipulation of the LSCA and reconstruction of the “antegrade” anatomical flow at the LSCA.

 In situ fenestration of endovascular stent grafts using laser, radiofrequency, and needle has been used for branched vessel revascularization [5–7]. Ohki et al. described a positive single-center experience of RIBS that had required zone 0 landing TEVAR [5]. Moreover, previous studies have reported the application of laser [6] and radiofrequency [7] techniques for in situ fenestration. Laser and radiofrequency catheters are more flexible and handle bending better than needle techniques; however, these energy-based devices have unique limitations. Tse et al. [7] reported that radiofrequency puncture of polytetrafluoroethylene was unsuccessful. Moreover, the interaction of laser with polytetrafluoroethylene graft materials may induce the formation of toxic substances [8]. Thus, laser and radiofrequency techniques may limit the types of stent graft compatible with in situ fenestration of endovascular stent grafts. The GORE TAG Thoracic Branch Endoprosthesis (TBE device; W. L. Gore & Associates, Inc., Flagstaff, AZ) is a good alternative [9]. However, branched devices manufactured by this company have limited anatomic variations and are not commercially available everywhere. The RIBS technique, in which the needle is simply bent for reconstruction of the LSCA, is a versatile and favorable alternative to these techniques in terms of graft selection and anatomic variations.

This case revealed the potential of two-stage repair of multiple thoracic aneurysms with right-sided vocal cord morbidity to prevent bilateral vocal cord injury. The use of the RIBS technique facilitated reconstruction of the LSCA by simply bending the needle. With the increase in surgical options to include endovascular and hybrid approaches, surgical procedures for complex aortic diseases should be carefully planned.

## Supplementary Information

**Supplementary Material 1.**

## Data Availability

The data supporting this study’s findings are available from the corresponding author on reasonable request.

## Abbreviations

TEVAR: Thoracic endovascular aortic repair
RIBS: Retrograde in situ branched grafting
CT: Computed tomography
RSCA: Right subclavian artery
BCA: Brachiocephalic artery
LSCA: Left subclavian artery
LCCA: Left common carotid artery
RCCA: Right common carotid artery
FET: Frozen elephant trunk
d-SINE: Distal stent graft-induced new entry
