# Supplementary material for: Two-staged hybrid repair of multiple great vessel and thoracic aneurysms with right vocal fold palsy using retrograde in situ branched stent grafting: a case report
Source: Gen Thorac Cardiovasc Surg Cases. 2024 Mar 4;3:18. doi: 10.1186/s44215-024-00142-w (PMC11533672; doi:10.1186/s44215-024-00142-w)
Supplement: Supplementary file 1 — Supplementary Material 1. [file 44215_2024_142_MOESM1_ESM.zip › Video_legends 2023.10.2R1.docx]

**Video Legend**

Whole procedures were shown in the Video clip.
